# I prefer what you can see: The role of visual perspective-taking on the gaze-liking effect

**DOI:** 10.1016/j.heliyon.2024.e29615

**Published:** 2024-04-16

**Authors:** Song Zhou, Yihan Sun, Yan Zhao, Tao Jiang, Huaqi Yang, Sha Li

**Affiliations:** a School of Psychology, Fujian Normal University, Fuzhou, China; b Huazheng School, Dongguan, China; c Research Center for Regional and National Comparative Diplomacy, China Foreign Affairs University, Beijing, China

**Keywords:** Gaze, Gaze-liking effect, Visual perspective-taking, Attention orientation, Mentalizing

## Abstract

Individuals' gaze on an object usually leads others to prefer that object, which is called the gaze-liking effect. However, it is still unclear whether this effect is driven by social factors (i.e., visual perspective-taking) or the domain-general processing (i.e., attention cueing). This research explored the mechanism of the gaze-liking effect by manipulating the objects' visibility to an avatar in six online one-shot experiments. The results showed that participants' affective evaluation for the object was modulated by the avatar's visual perspective. Specifically, the visible object to the avatar received a higher rating of liking degree. However, when the avatar was replaced with a non-social stimulus, the experimental effect was absent. Furthermore, the gaze-liking effect was robust while controlling for confounding factors such as the distance between the object and the avatar or type of stimuli. These findings provided convincing evidence that the gaze-liking effect involves a process of the other's visual experience and is not merely a by-product of the gaze-cueing effect.

## 1 Introduction

Gaze plays an essential mediate role in inferring others' intention within human's social environment [1,2], and the interpretation of other's gaze is a crucial factor in successful cooperation and joint action. For example, gaze could orientate observers' attention, which is known as the gaze-cueing effect [3]. Individuals can perceive where others are looking at by tracing others' gaze, which can draw their attention to the corresponding spatial location [[4], [5], [6]]. Though it has been argued that humans might process gaze just as a directional symbol like arrows, research has shown that the gaze-cueing effect can be shaped by several social factors, including mental state attribution [7]. Interestingly, one's subjective evaluation of the object, except for their attention, could also be affected by the person gazing at it. It has been found that one's gaze can influence others' affective judgement for the target, which is known as the gaze-liking effect, where people are likely to prefer the object that is being gazed by others [8]. The gaze-liking effect is widely applied in the design of commercials to build up the audience's' positive impression of the displaying products. However, since the gaze is associated with both domain-general attention orientation and social cognitive process, the mechanism by which the gaze-liking effect is driven is still under debate.

The mechanism of gaze process is rooted in two main views [9]. Since the gaze can serve as an attentional cue that directs one's attention to a particular spatial location, some argue that the gaze-liking effect is driven by a domain-general process and attributed to the attention-cueing effect. For instance, participants were found to rate a higher preference for the target pointed by non-social cues, such as arrows [[10], [11], [12]]. Other researchers believe that the gaze-liking effect is caused by a long exposure time as a result of directed attention [13,14]. In this context, the gazes of others may direct a person's attention to a specific object, which increase their exposure time to the object and consequently increase their degree of like; in turn, this further increase the amount of attention they give to the object. Ultimately, this positive feedback cycle produces a gaze-liking effect.

However, there are also some authors who emphasize the role of the social attributes of gaze [[15], [16], [17]], suggesting that the gaze-liking effect occurs through social cognitive processes. The features of the agent, especially the emotional characteristics, were found significantly affect the gaze-liking effect. For example, the agent's expression is thought to shape the gaze-liking effect. Particularly, when a face gazes at an object with an expression of disgust, such object tends to receive a lower degree of preference from observers [18,19]. Moreover, the trustworthiness of the agent's face may impact the extent to which observers like the target. If the agent is perceived as trustworthy, the object they are gazing at should gain a higher degree of liking. By contrast, the gaze-liking effect is not found when the agent is perceived as untrustworthy [20,21]. In addition, the attractiveness of the agent's face is considered to affect the degree to which others like the corresponding object [22]. While the gaze may orient participants' attention to the target, the characteristics of the agent, however, may also influence participants' internal experiences, which can affect their preference for the target consequently.

A more straightforward way to examine the social cognitive account of the gaze-liking effect is to investigate the role visual perspective-taking plays in this effect. Visual perspective-taking is considered as a fundamental aspect of mentalizing as it involves perceiving an object's visibility to an agent, which is a higher level of processing than identifying the object's spatial location [23,24]. Following this idea, Manera et al. revealed that the gaze-liking effect was also influenced by the visibility of the object to the agent [25]. After replicating the basic gaze-liking effect, they conducted two experiments to examine whether this effect is driven by seeing rather than merely looking. The authors expected that the participants would have an impression that a face was able (or not able) to see the object by manipulating the way objects were presented in terms of time. They found that the gaze-liking effect could be observed when the object emerged with the face got gradually occluded by panels. However, the effect was absent when the object appeared suddenly after the face was totally occluded by panels. Manera et al. argued that the face was supposed to be unable to see the object when the object appeared from nowhere. This result implies that the gaze-liking effect is achieved through visual perspective-taking rather than attention-orientation. However, the paradigm that Manera et al. used has several limitations. The ability for the face to see the object was manipulated by the relative time they were presented; authors assumed that participants would consider the face was not able to see the object when the face and object were not simultaneously appeared. Nonetheless, humans' inference for others' visual experiences were basically based on spatial rather than temporal information, making the manipulation in Manera et al.’s study not intuitive. In particular, not presenting the face and objects at the same time may weaken participants' taking of the face's visual perspective. Besides, there were many factors that might affect the gaze-liking effect in their study, such as the speed of the panel's movement, and the duration of the face's presentation. As a result, these limitations might weaken the persuasiveness of their findings.

In summary, there is no consensus on the role of the gaze in the gaze-liking effect currently. On one hand, studies suggest that the gaze is a domain-general attention cue often but cannot completely exclude its social influence [9]; on the other hand, the introduction of confounding factors support the role of gaze as a social cue inducing a more complex interpretation of the results. More importantly, a pre-registered study replicated the original study of the gaze-liking effect with a larger sample size reported a small-to-null effect size of the gaze-liking effect [26], challenging the validity of the gaze-liking effect. While the study conducting the same paradigm as Bayliss', examining the gaze-liking effect with new paradigm might help resolve these issues.

The present study explored the role of the gaze in the gaze-liking effect by referring to the classical experimental paradigm of visual perspective-taking, the dot-perspective task (DPT) [27]. Under this framework, we aimed to eliminate confounding factors and provide reliable evidence on the actual role of the gaze in this unresolved problem. Additionally, the utilization of DPT makes it possible to investigate the role that visual perspective-taking plays on the spatial dimension. Specifically, the visibility of objects is manipulated by blocking (or not blocking) the agent's line of view. We conducted six experiments to explore the mechanism behind the gaze-liking effect. Notably, the current study conducted a series of one-shot online experiments. In the field of visual perspective-taking studies, one-shot experiments are accepted widely when the study does not focus on the response time [[28], [29], [30]]. Additionally, the present study concerned itself more with the participants' liking degrees for the object, which is an intuitive judgement that can be better reflected by a single trial. In our experiments, an avatar stands sideways in the center of the scene, and several letters serve as targets for participants to rate their degree of preference. The visibility of the letters to the avatar was manipulated by their position relative to the agent (in front of or behind the avatar) and whether the avatar's line of sight to the letter was blocked by a barrier. If participants rated a higher liking degree for the letter visible to the avatar, then it would support that the gaze-liking effect is driven by visual perspective-taking.

In addition, we directly examined whether the gaze-liking effect is merely a product of the attention-cueing effect in the last experiment. Regarding the DPT, studies have shown that non-social stimuli such as arrows and fans could yield the same consistency effect as the human-like avatar [31,32]. With reference to the work of Vestner et al. we used a fan instead of the avatar as a stimulus in Experiment 6 to further investigate whether the social attribution of the stimulus is the factor of producing the gaze-liking effect.

## 2 Experiment 1

To test whether the gaze-liking effect is driven by visual perspective taking, we designed a stimulus image based on the DPT in which the avatar can see only one of the objects [27]. In Experiment 1, we positioned two letters on the same side of the avatar, then manipulated the visibility of those letters to the agent by setting barriers between the letters and the agent. Since both letters were positioned in the facing direction of the avatar, their spatial location would be cued by the avatar's orientation. However, if the gaze-liking effect was associated with a mentalizing process, then the visible letter should be evaluated as more likable.

### 2.1 Method

***Participants.*** An identical number of 144 participants were recruited across six experiments, and the sample size was determined for the following reasons. In the present study, the only factor that was analyzed was the visibility of the target to the agent (visible vs. invisible). However, the stimulus image was created according to the arrangement of the scene, resulting in different numbers of stimuli images across six experiments, and one image corresponded to one trial. Since the present study was a one-shot experiment that each participant completed only one trial, we determined the sample size based on 1) a prior statistical analysis and 2) the integral multiple of the number of images that experiment created. The prior statistical analysis using G*Power 3.1 demonstrated that a minimum sample size of 137 (with effect size *d* = 0.30 according to a previous study [33], alpha = 0.05) was required to reach a power of 0.95 for a paired sample *t*-test [34], and the nearest integral multiple of the number of images is 144.

We initially recruited 50 redundant participants for each trial, among which the first 36 (144 participants divided by 4 images) non-outlier responses (defined as completing the trial within the mean completing time ± 2 SD) were included in the formal analysis. Finally, 144 valid participants' data (57 males, 87 females; average age of 28.45 ± 12.31) were included in Experiment 1. All participants provided informed consent in accordance with the Declaration of Helsinki and received 2 yuan after they finish the task as remuneration. No personal information that can identify individual participants was collected during the study. The experimental protocol was approved by the Institutional Review Board of [redacted for peer review], approval number [redacted for peer review].

***Material & Procedure.*** All experiments in this study were conducted in 2022 (except for Experiment 6 which is conducted in 2023) on an online survey platform. Participants were informed to view an image of experimental scene after providing demographic information, and then rate their preferences for all the targets presented in the image.

**Fig. 1:**
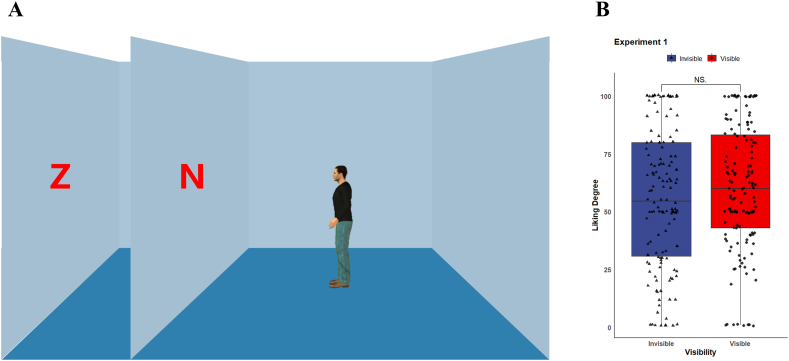
(A) Example of image of scene in Experiment 1. (B) Average liking ratings for the letters.

The experimental scene was adapted from the DPT, which used to examine the effect of visual perspective taking [27,35,36]. In simple terms, this paradigm manipulated the visibility of the targets to the agent to investigate whether participants' performances were affected by the manipulation. In the scene of the DPT, an avatar is standing sideways (left- or right-orientation) in the center of a room, and targets might be present on the wall in front of or behind the avatar. Besides, a barrier (e.g., another wall) might be set in front of the avatar to block their line of view to the target. We modified the classical scene of the original DPT to compatible the gaze-liking effect study. Specifically, we choose 2 English letters (i.e., “N” and “Z”) as the target that be present on the walls in front of the avatar. However, only one of the two letters was visible to the avatar (see Fig. 1A). Participants were requested to view one stimulus image and then rate their liking degree for the two letters by dragging a slider on a scale ranging from 1 to 100. There is no time limitation to complete the rating, but those completing it for longer than the mean time plus 2 SD were excluded from the analysis.

In total of 4 stimulus images were created based on the letter arrangement (“ZN” or “NZ”) and avatar's orientation (left or right). All letters were present in the same size of 56 × 97 pixels, and the orientation of the avatar and the positions of the letters were counterbalanced across the participants. We conducted a one-shot online experiment so that each image was viewed by 36 participants.

### 2.2 Results and discussion

We implemented a paired sample *t*-test to examine participants’ preference for the letters. The result demonstrated the visible letter (*M* = 60.3, *SD* = 26.8) obtained a slight higher liking rate than the invisible letter (*M* = 55.6, *SD* = 29.8), while the effect of letter visibility was not significant, *t*(143) = 1.526, *d* = 0.127, *p* = 0.129, see Fig. 1B.

Surprisingly, the gaze-liking effect was not observed in Experiment 1. Since both letters were presented in front of the agent, it is possible that the gaze-liking effect is driven by attention-orientation. However, considering that the effect of attention-orientation was not directly examined in Experiment 1, it is too early to be conclusive. For example, a third letter presented on the wall behind the agent is needed to examine the effect of attention-orientation.

Nonetheless, there was also possibility that the gaze-liking effect was driven by visual perspective-taking, yet participants failed to take the agent's visual perspective spontaneously. Currently, the spontaneity of visual perspective-taking is still an open question. Albeit the consistency effect was found even when participants were not instructed to adopt the avatar's visual perspective in the original study of Samson et al. subsequent research challenged the argument of the spontaneity of visual perspective-taking. For example, a similar pattern could also be observed when using a non-social stimulus or when the avatar's line of sight is blocked [32,37,38]. Therefore, we explicitly asked participants to consider the avatar's visual experience in Experiment 2 to ensure the involvement of visual perspective-taking processing in the task.

## 3 Experiment 2

**Fig. 2:**
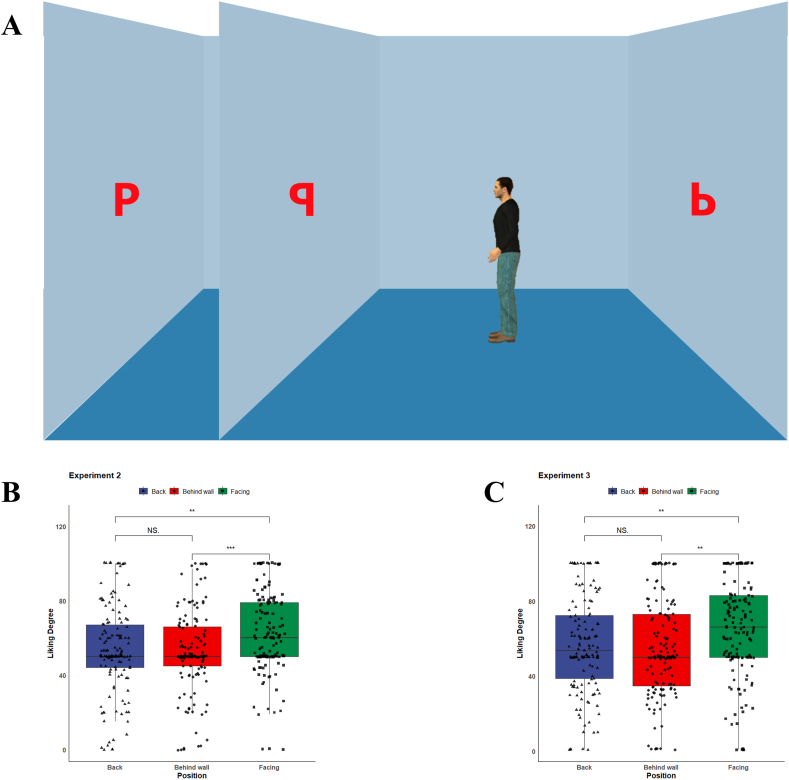
(A) Example image for Experiment 2 and 3, and (B) average liking ratings for letters in Experiment 2 and (C) Experiment 3. **p < 0.01, ***p < 0.001.

According to the results of Experiment 1, we made two improvements in Experiment 2. The first was to add another letter to the wall behind the agent (see Fig. 2A), the addition of which could more effectively verify whether the gaze-liking effect is driven by the domain-general attention-cueing. The second was to add a question asking which letter the agent could see after participants viewed the image, thus inducing them to explicitly take the avatar's visual perspective.

### 3.1 Method

***Participants.*** One hundred and forty-four participants (42 males, 102 females; average age of 27.63 ± 13.67 years) having no prior knowledge of the present study were recruited online under the same criteria as Experiment 1.

***Material & Procedure.*** We modified the experimental scene based on the result of Experiment 1. Compared to the scene of Experiment 1, a third letter was present on the wall behind the agent In Experiment 2. The avatar could only see the letter in the middle. To avoid the potential effect of the letters' physical features, three letters (q, d, and p) with similar appearance were used in Experiment 2. Consequently, a total of 6 (letter arrangement: pdq, pqd, dpq, dqp, qpd, qdp) × 2 (avatar orientation: left, right) = 12 images of scenes were produced. Additionally, a question was asked of the participants as to which letter the agent could see in the image in order to further prime them to take the agent's visual perspective. The question was presented before the participants rated their preferences. The rest of experimental design was identical to Experiment 1.

### 3.2 Results and discussion

We excluded the data from the participants those who answered incorrectly to the question about the visibility of the letters to the agent. A repeated measures ANOVA showed that the effect of positions of the letters on the liking degree was significant (*F*(2, 143) = 9.44, *p* < 0.001, η^2^ = 0.062). As demonstrated by the post-hoc test, the degree of preference for the visible letter (*M* = 62.3, *SD* = 22.1) was significantly higher than that of the other two letters (with the letter behind the wall, *M* = 52.8, *SD* = 22.3; *t*(143) = 4.007, *d* = 0.670, *p* < 0.001; with the letter behind the agent, *M* = 53.7, *SD* = 23.7; *t*(143) = 3.405, *d* = 0.569, *p* = 0.002). The difference between the two invisible letters was not significant, *t*(143) = 0.373, *d* = 0.062, *p* = 0.926, see Fig. 2B.

A significant gaze-liking effect was observed as the participants were instructed to take the agent's visual perspective. Participants rated their preferences for the only letter visible to the avatar higher than any of other letters, while their preferences for the other two letters showed no difference. This suggests that the attention-orientation effect of the avatar did not trigger the gaze-liking effect by itself. If the attention-orientation effect accounted for the gaze-liking effect, participants would rate their degree of liking for the two letters in front of the agent at more or less the same level. Rather, the result implied that the gaze-liking effect occurred only when people knew clearly what others were gazing at.

However, the question we added to encourage participants to take the agent's visual perspective may also introduced confounding variable. Specifically, participants were asked which letter the avatar could see, and the correct answer was one particular letter. This may lead participants pay more attention to and have incidentally increased familiarity with the particular letter, thus affecting how participants judged their liking degree in the subsequent question [11,39,40]. Experiment 3 was conducted to exclude the potential explanation.

## 4 Experiment 3

### 4.1 Method

***Participants.*** We recruited 144 valid participants (36 males, 108 females; average age of 28.51 ± 14.2 years). The participants were naïve to the purpose of Experiment 3. The remaining was the same as in Experiment 2.

***Material & Procedure.*** The only difference between Experiments 2 and 3 is that we asked three questions about the visibility of each of the three letters instead of asking which letter the avatar could see. The rest of the experiment was identical to Experiment 2.

### 4.2 Results and discussion

A repeated measures ANOVA demonstrated that the difference in liking degree among the three positions of the letters was significant (*F*(2, 143) = 7.582, *p* < 0.001, η^2^ = 0.050). Specifically, the liking degrees for letters visible to the agent (*M* = 64.6, *SD* = 25.9) were significantly higher than those for other letters, i.e., the letters behind the avatar (*M* = 56.5, *SD* = 25.9; *t*(143) = 3.256, *d* = 0.544, *p* = 0.004) and letters behind the wall (*M* = 55.0, *SD* = 26.3; *t*(143) = 3.390, *d* = 0.567, *p* = 0.003). The difference in liking degree between the two invisible letters was not significant, *t*(143) = 0.561, *d* = 0.093, *p* = 0.841, see Fig. 2C.

The results revealed a similar pattern of Experiment 2, which support the social cognitive account of the gaze-liking effect. Considering that the visibilities of all three letters were asked, it could be inferred that any difference in familiarity induced by the question in Experiment 2 was not the main cause for the gaze-liking effect. On the contrary, this was exactly the effect induced by visual perspective taking, as verified in Experiment 3. In this condition, only objects within the avatar's visual perspective is given higher liking degrees.

However, due to the design of the stimulus images, the visible letter was constrained as the closest letter to the avatar in all conditions. This might introduce a confounding variable, such as the distance between the letters and the avatar. We revised the stimulus images to make the closest letter invisible to the avatar in Experiment 4 to address this issue.

## 5 Experiment 4

### 5.1 Method

***Participants.*** A total of 144 new participants (57 males, 87 females; average age of 23.78 ± 5.43 years) was recruited online. The remaining was the same as in Experiment 2.

**Fig. 3:**
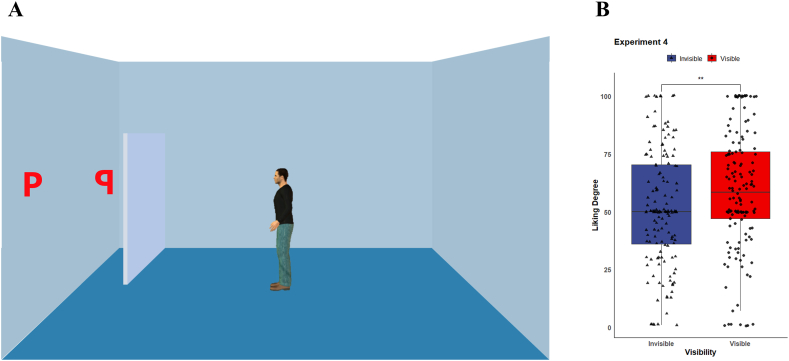
(A) Example of image of scene in Experiment 4. (B) Average liking ratings for the letters.

***Material & Procedure.*** Experiment 4 was conducted to exclude the confounding factor of distance between the letters and the avatar. Since we had found that the difference in liking degree between the two invisible letters was not significant, Experiment 4 focused on the two letters in front of the avatar. We modified the position of the barrier so that from the perspective of the participant, the invisible letter was closer to the avatar, which was contrary to the stimulus images of Experiment 3 (see Fig. 3A). There was a total of 2 (letter arrangement: pq, qp) × 2 (avatar orientation: left, right) = 4 stimulus images created. Before participants rated their liking for the two letters, they were asked whether the avatar could see each of the two letters. The conditions were balanced between participants.

### 5.2 Results and discussion

We implemented a paired sample *t*-test to examine participants’ preference for the letters. The result demonstrated the liking degree for the visible letter (*M* = 58.8, *SD* = 25.6) was significantly higher than the invisible letter (*M* = 51.7, *SD* = 25.4), *t*(143) = 2.769, *d* = 0.230, *p* = 0.006, see Fig. 3B.

We observed a significant gaze-liking effect. Participants preferred the visible letters of the avatar over the invisible letters of the avatar, even though the invisible letters were closer to the avatar and in the center of the visual field. This suggests that the gaze-liking effect was not due to distance or attentional accounts but may be explained by the participants' visual perspective taking, which is what triggered the gaze-liking effect for visible letters.

Even though we found the above results on letters, we have not yet verified this gaze-liking effect on real objects, which may be more meaningful and important. Therefore, Experiment 5 we will do further validation on real objects.

## 6 Experiment 5

### 6.1 Method

***Participants.*** Similar to Experiment 4, 144 new participants (66 males, 78 females; average age of 26.71 ± 8.62 years) with an identical criterion were enrolled online for Experiment 5.

**Fig. 4:**
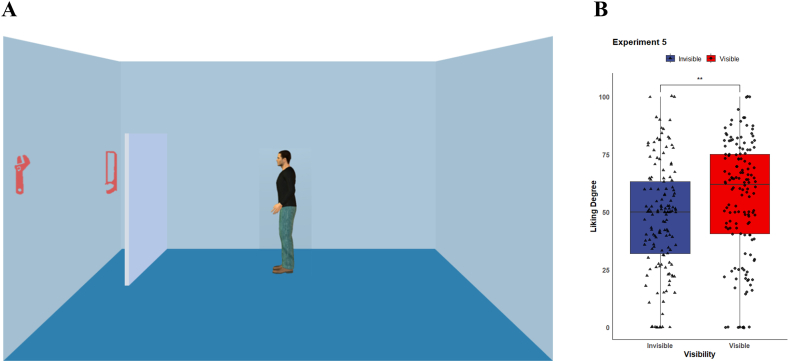
(A) Example of image of scene in Experiment 5. (B) Average liking ratings for the tools.

***Material & Procedure.*** The stimuli images of Experiment 5 were similar to Experiment 4, except that the two letters were replaced with four icons of household tools (e.g., wrench and saw; see Fig. 4A). The icons were adopted from the original work of Bayliss [18]. Hence, we created 24 stimulus images for Experiment 5, and each participant viewed two images (avatar facing right and left) in Experiment 5. The rest of the experiment was the same as Experiment 4.

### 6.2 Results and discussion

We excluded the data from the participants those who answered incorrectly to the question about the visibility of the objects to the avatar. We implemented a paired sample *t*-test to examine participants' preference for the objects. The result demonstrated the effect of object visibility on the liking degree was significant (see Fig. 4B), *M*_visible_ = 56.0, *SD*_visible_ = 25.1; *M*_invisible_ = 47.4, *SD*_invisible_ = 23.9; *t*(143) = 3.546, *d* = 0.353, *p* = 0.001.

The results showed a similar pattern to Experiment 4, in which we found the same gaze-liking effect for objects visible on real objects. Participants showed a higher preference for visible objects at farther distances. This further validates the validity of the results of Experiment 4, which is exactly the gaze-liking effect induced by visual perspective.

## 7 Experiment 6

Finally, we conducted Experiment 6, inspired by Vestner et al.'s work, to examine the role of attention cueing in the gaze-liking effect. If the gaze-liking effect could be triggered by the attention-cueing effect, a non-social stimulus with a clear orientation should produce the same effect as well. We replaced the avatar in the stimulus image with a fan which has a clear orientation as the avatar so that participants' attention could be shifted.

### 7.1 Method

***Participants.*** We recruited 144 new participants (41 males, 103 females; average age of 21.33 ± 2.75 years) with the identical criterion as the preceding experiments for Experiment 6.

**Fig. 5:**
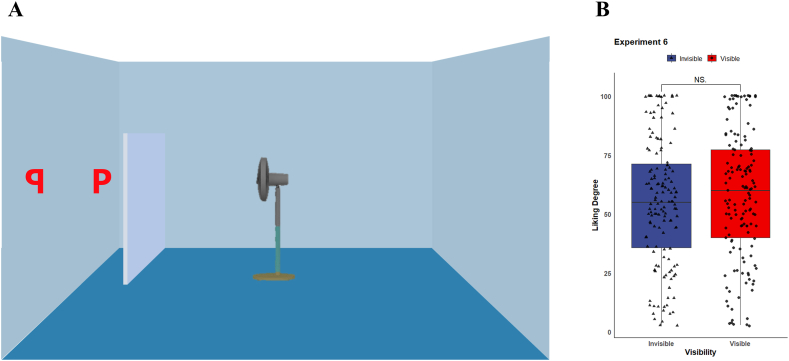
(A) Example of image of scene in Experiment 6. (B) Average liking ratings for the letters.

***Material & Procedure.*** The stimuli images of Experiment 6 were similar to Experiment 4, but the avatar in the center of the room was replaced with a fan. We adjust the color of the fan to make it visually match the image of the agent (see Fig. 5A). Moreover, to rule out the possibility that the visibility-judgement question might draw additional attention to the only visible letter and hence produce the effect, we asked a similar question in Experiment 6 that which letter could get blown by the fan.

### 7.2 Results and discussion

As we replaced the avatar with a fan in Experiment 6, the liking effect was absent, where *M*_visible_ = 58.0, *SD*_visible_ = 27.1; *M*_invisible_ = 55.4, *SD*_invisible_ = 27.0; *t*(143) = 0.829, *d* = 0.069, *p* = 0.409 (see Fig. 5B). Since the fan has an explicit orientation, it could exert a comparable attention-cueing effect as an avatar could. However, in this scenario, there is no visual perspective for participants to adopt. The negative result in Experiment 6 further illustrated that the adoption of other's visual perspective might be the crucial factor to produce the gaze-liking effect.

## 8 General discussion

This study implemented a revised dot-perspective task paradigm to explore the mechanism behind the gaze-liking effect. We found that the gaze-liking effect observed in previous studies not only required the gaze, but also needed to meet two other conditions. First, individuals must take the visual perspective of the avatar to understand what is visible therein. Second, any corresponding object must fall within the visual field of the avatar. In the absence of either condition, the gaze-liking effect will not be activated.

To reach this conclusion, we conducted six experiments. These results verified that visual perspective-taking is necessary for the gaze-liking effect. Thus, we obtained a preliminary answer to the question raised in the introduction; that is, to produce the gaze-liking effect, the gaze plays the role as a social cue (rather a simple attention cue) that requires a mentalizing process (i.e., visual perspective taking). However, this result is not completely accurate. Many previous studies have explored the gaze effect by searching for special features, as compared to other attention-oriented cues [41,42]. In fact, the real issue may be visual perspective-taking. The most important difference between the gaze and other non-social attention cues may be that the gaze more strongly motivates observers to take its visual perspective spontaneously [30,36], which can trigger the liking effect. As this study found, the liking effect does not occur when individuals observe the gaze but do not take its perspective. Such a phenomenon is not truly a “gaze-liking effect,” but can more suitably be termed a “visual perspective-liking effect.”

In Experiment 1, we positioned both letters on the same side of the agent, then altered the visibility of those letters to the agent by setting barriers. In Experiment 2, we addressed this concern by placing an additional letter behind the agent as a control condition. Presented with this arrangement, participants were asked which letter the agent could see, then instructed to report their degree of liking. This visibility question aimed to increase the potential that participants would take the avatar's visual perspective. Whether the letter was blocked by the barrier in front of the agent or located behind the agent, its degree of liking was significantly lower than that for letters the agent could see, meaning that the gaze-liking effect was based on visual perspective taking. In Experiment 3, we eliminated the potential cuing effect from the question, and still produced results that were consistent with Experiment 2. This again verified the important role of visual perspective-taking in the gaze-liking effect. In addition, the validity of this effect was further examined in Experiment 4 and 5, excluding the potential effect of confounding factors. Indeed, the visibility-judgement questions we asked before the preference rating may produce the primming effect. However, as illustrated in Experiment 4 and 5, all target objects were presented in front of the avatar, yet only the visible object was rated as more likable. Additionally, the results of Experiment 4 demonstrated that the relative spatial relationship would not affect the gaze-liking effect. The letter that receives the highest preference rating is always the one that is visible, but not the one closest to the avatar.

More importantly, no effect was found in Experiment 6, in which the avatar was replaced with a fan. Even though it has been proved that the fan could orient attention in the DPT [30], such spatial-based attention shifting is distinctive from the mentalizing processing of visual perspective-taking. The negative result of Experiment 6 suggests that social attribution might be the crucial factor in producing the gaze-liking effect. Especially since we have asked participants visibility-like questions in Experiment 6, it seems that this kind of question would not cause additional attention to the particular letter, otherwise the additional attention is unable to produce the gaze-liking effect. Albeit there is a possibility that the visibility question brings about additional attention to the only visible letter in the preceding experiments, it is worth discussing whether this attention could be thought as pure perceptual processing. In fact, any additional attention to the visible letter was achieved based on the inference of the avatar's visual experience, implying that the visual perspective-taking was the basic reason for the gaze-liking effect.

Compared to the original DPT paradigm [27], the present study revised the experimental procedure. Participants in the original DPT were specifically asked to adopt their own or the avatar's visual perspective before responding. The authors discovered that the participants' responses were influenced by the conflict between their own perspective and the avatar's perspective, even though there was no instruction to consider the avatar's visual perspective, which is referred to the consistency effect. However, whether the consistency effect in the self-perspective condition could be thought to evidence the spontaneity of the visual perspective-taking is still under debated. In the present study, in order to ensure the presence of visual perspective-taking processing, participants were requested to only take the avatar's visual perspective. Yet, this discrepancy would not affect the interpretation of the main results of the present study since we did not concern with the spontaneity of the visual perspective-taking. It matters, in the present study, whether participants' affective evaluations for the objects were affected by the visibility of objects to the avatar. Therefore, though participants were not instructed to take their own perspective as the original DPT, our results demonstrated that the adoption of the avatar's visual perspective is crucial for the gaze-liking effect.

Our results may also provide a reasonable explanation for inconsistent research conclusions in the past. Since the gaze-liking effect was proposed by Bayliss et al., in 2006 [8], there have been persistent debates over the driven mechanism of the effect. Especially, Tipples and Pecchinenda implemented an identical paradigm that used by Bayliss et al. and found a much smaller effect size than the original study [26]. Similar to the original study, they used 36 images of household items as target objects and conduct 432 trials for every participant. However, repeatedly exposure to the same item may affect individuals' subjective attitude towards it, especially when involved in preference ratings. Besides, participants in their study were requested to rate their liking for one specific item per trial, we instructed participants to rate for both visible and invisible items simultaneously, such a contrast could better represent the difference in liking-rating between the items and exert a clearer gaze-liking effect. Therefore, we believe that the paradigm we employed is more appropriate for examine the role visual perspective-taking plays in the gaze-liking effect.

It should be noted that there are limitations that may weaken the validity of our results. Firstly, the form of one-shot experiment conducted in the present study is a controversial method to explore human's behavioral response. Although one-shot experiments have been implemented in several visual perspective-taking studies, some argue that these results should be inspected under the classical experimental method. Therefore, a replication of our study with the classical experimental method might help to enhance the convincingness of our results. Secondly, the result of Experiment 6 might be false-negative due to the particular design of the stimulus images. Because the wall in the scene could not fully partition the room, participants might doubt that both letters could get blown by the fan, even in an indirect way. More studies should be employed to carefully design stimulus images to further confirm the role of purely perceptual attention cueing in the gaze-liking effect.

## 9 Conclusion

We conducted six experiments to investigate the role of visual perspective-taking in the gaze-liking effect and found that this effect occurred only when the participants were conscious what the agent could see. Through our research, we have gained a deeper understanding of the mechanism underlying the gaze-liking effect. Our findings suggest that the gaze-liking effect is a phenomenon driven by mentalizing processes, i.e., visual perspective-taking, rather than merely a domain-general attention orientation.

## Funding

This study is supported by 10.13039/501100003392Natural Science Foundation of Fujian Province (2022J05045).

## Ethics statement

This study was reviewed and approved by Institutional Review Board of School of Psychology, [redacted for peer review], with the approval number PSY220024. All participants provided informed consent to participate in the study.

## Data availability statement

All data produced during the current study are available on the Open Science Framework (https://osf.io/2e65t/).

## CRediT authorship contribution statement

**Song Zhou:** Methodology, Funding acquisition, Formal analysis, Data curation, Conceptualization. **Yihan Sun:** Validation, Resources, Formal analysis. **Yan Zhao:** Resources, Investigation. **Tao Jiang:** Writing – review & editing, Investigation. **Huaqi Yang:** Writing – review & editing, Writing – original draft, Visualization, Formal analysis, Conceptualization. **Sha Li:** Writing – original draft, Investigation.

## Declaration of competing interest

The authors declare that they have no known competing financial interests or personal relationships that could have appeared to influence the work reported in this paper.

## References

[1] Hessels R.S. (2020). How does gaze to faces support face-to-face interaction? A review and perspective. Psychon. Bull. Rev..

[2] Hietanen J.O., Peltola M.J., Hietanen J.K. (2020). Psychophysiological responses to eye contact in a live interaction and in video call. Psychophysiology.

[3] Frischen A., Bayliss A.P., Tipper S.P. (2007). Gaze cueing of attention: visual attention, social cognition, and individual differences. Psychol. Bull..

[4] McKay K.T., Grainger S.A., Coundouris S.P., Skorich D.P., Phillips L.H., Henry J.D. (2021). Visual attentional orienting by eye gaze: a meta-analytic review of the gaze-cueing effect. Psychol. Bull..

[5] Riechelmann E., Gamer M., Böckler A., Huestegge L. (2021). How ubiquitous is the direct-gaze advantage? Evidence for an averted-gaze advantage in a gaze-discrimination task. Atten. Percept. Psychophys..

[6] Zhang J., He X., Sommer W., Yue Z. (2021). Does gaze direction of fearful faces facilitate the processing of threat? An ERP study of spatial precuing effects. Cognit. Affect Behav. Neurosci..

[7] Dalmaso M., Castelli L., Galfano G. (2020). Social modulators of gaze-mediated orienting of attention: a review. Psychon. Bull. Rev..

[8] Bayliss A.P., Paul M.A., Cannon P.R., Tipper S.P. (2006). Gaze cuing and affective judgments of objects: I like what you look at. Psychon. Bull. Rev..

[9] Capozzi F., Ristic J. (2020). Attention AND mentalizing? Reframing a debate on social orienting of attention. Vis. Cognit..

[10] Callejas A., Shulman G.L., Corbetta M. (2014). Dorsal and ventral attention systems underlie social and symbolic cueing. J. Cognit. Neurosci..

[11] Tipples J., Dodd M., Wickstrom J., Kingstone A. (2019). Verbal Descriptions of cue direction affect object desirability. Front. Psychol..

[12] Besner D., McLean D., Young T. (2021). On the determination of eye gaze and arrow direction: Automaticity reconsidered. Canadian Journal of Experimental Psychology/Revue canadienne de psychologie expérimentale.

[13] Shimojo S., Simion C., Shimojo E., Scheier C. (2003). Gaze bias both reflects and influences preference. Nat. Neurosci..

[14] Li Z.-W., Ma W.J. (2021). An uncertainty-based model of the effects of fixation on choice. PLoS Comput. Biol..

[15] Gobel M.S., Tufft M.R., Richardson D.C. (2018). Social beliefs and visual attention: how the social relevance of a cue influences spatial orienting. Cognitive science.

[16] Wang N., Xu S., Zhang S., Luo Y., Geng H. (2019). ERP evidence on how gaze convergence affects social attention. Sci. Rep..

[17] Colombatto C., Chen Y.-C., Scholl B.J. (2020). Gaze deflection reveals how gaze cueing is tuned to extract the mind behind the eyes. Proc. Natl. Acad. Sci. USA.

[18] Bayliss A.P., Frischen A., Fenske M.J., Tipper S.P. (2007). Affective evaluations of objects are influenced by observed gaze direction and emotional expression. Cognition.

[19] Kaisler R.E., Marin M.M., Leder H. (2020). Effects of emotional expressions, gaze, and head orientation on person perception in social situations. Sage Open.

[20] King D., Rowe A., Leonards U. (2011). I trust you; hence I like the Things you look at: gaze cueing and sender trustworthiness influence object evaluation. Soc. Cognit..

[21] Barbato M., Almulla A.A., Marotta A. (2020). The effect of trust on gaze-mediated attentional orienting. Front. Psychol..

[22] Kaisler R.E., Leder H. (2016). Trusting the looks of others: gaze effects of faces in social settings. Perception.

[23] Ward E., Ganis G., Bach P. (2019). Spontaneous vicarious perception of the content of another's visual perspective. Curr. Biol..

[24] Ward E., Ganis G., McDonough K.L., Bach P. (2020). Perspective taking as virtual navigation? Perceptual simulation of what others see reflects their location in space but not their gaze. Cognition.

[25] Manera V., Elena M., Bayliss A., Becchio C. (2014). When seeing is more than looking: intentional gaze modulates object desirability. Emotion.

[26] Tipples J., Pecchinenda A. (2019). A closer look at the size of the gaze-liking effect: a preregistered replication. Cognit. Emot..

[27] Samson D., Apperly I., Braithwaite J., Andrews B., Scott S. (2010). Seeing it their way: evidence for rapid and involuntary computation of what other people see. J. Exp. Psychol. Hum. Percept. Perform..

[28] Martin R., Kusev P., van Schaik P. (2021). Autonomous vehicles: how perspective-taking accessibility alters moral judgments and consumer purchasing behavior. Cognition.

[29] Samuel S., Hagspiel K., Eacott M.J., Cole G.G. (2021). Visual perspective-taking and image-like representations: we don't see it. Cognition.

[30] Zhao X., Malle B.F. (2022). Spontaneous perspective taking toward robots: the unique impact of humanlike appearance. Cognition.

[31] Vestner T., Balsys E., Over H., Cook R. (2022). The self-consistency effect seen on the Dot Perspective Task is a product of domain-general attention cueing, not automatic perspective taking. Cognition.

[32] Santiesteban I., Catmur C., Hopkins S.C., Bird G., Heyes C. (2014). Avatars and arrows: implicit mentalizing or domain-general processing?. J. Exp. Psychol. Hum. Percept. Perform..

[33] Ulloa J.L., Marchetti C., Taffou M., George N. (2015). Only your eyes tell me what you like: exploring the liking effect induced by other's gaze. Cognit. Emot..

[34] Faul F., Erdfelder E., Buchner A., Lang A.-G. (2009). Statistical power analyses using G* Power 3.1: tests for correlation and regression analyses. Behav. Res. Methods.

[35] Baker L.J., Levin D.T., Saylor M.M. (2016). The extent of default visual perspective taking in complex layouts. J. Exp. Psychol. Hum. Percept. Perform..

[36] O'Grady C., Scott-Phillips T., Lavelle S., Smith K. (2020). Perspective-taking is spontaneous but not automatic. Q. J. Exp. Psychol..

[37] Langton S.R.H. (2018). I don't see it your way: the dot perspective task does not gauge spontaneous perspective taking. Vision.

[38] Cole G.G., Atkinson M., Le A.T.D., Smith D.T. (2016). Do humans spontaneously take the perspective of others?. Acta Psychol..

[39] Hansen J., Wänke M. (2009). Liking what's familiar: the importance of unconscious familiarity in the mere-exposure effect. Soc. Cognit..

[40] Mrkva K., Van Boven L. (2020). Salience theory of mere exposure: relative exposure increases liking, extremity, and emotional intensity. J. Pers. Soc. Psychol..

[41] Sato W., Kochiyama T., Uono S., Yoshikawa S. (2009). Commonalities in the neural mechanisms underlying automatic attentional shifts by gaze, gestures, and symbols. Neuroimage.

[42] Atkinson M.A., Simpson A.A., Cole G.G. (2018). Visual attention and action: how cueing, direct mapping, and social interactions drive orienting. Psychonomic Bull. Rev..

